# Is there really a proportional relationship between VO_2max_ and body weight? A review article

**DOI:** 10.1371/journal.pone.0261519

**Published:** 2021-12-21

**Authors:** Jay Lee, Xiuli Zhang

**Affiliations:** School of Physical Education & Sports Science, South China Normal University, Guangzhou, China; All India Institute of Medical Sciences, INDIA

## Abstract

Maximum oxygen uptake (VO_2max_) is a “gold standard” in aerobic capacity assessment, playing a vital role in various fields. However, ratio scaling ($\frac{VO_{2max}}{bw}$), the present method used to express relative VO_2max_, should be suspected due to its theoretical deficiencies. Therefore, the aim of the study was to revise the quantitative relationship between VO_2max_ and body weight (bw). Dimensional analysis was utilized to deduce their theoretical relationship, while linear or nonlinear regression analysis based on four mathematical models (ratio scaling, linear function, simple allometric model and full allometric model) were utilized in statistics analysis to verify the theoretical relationship. Besides, to investigate the effect of ratio scaling on removing body weight, Pearson correlation coefficient was used to analyze the correlation between $\frac{VO_{2max}}{bw}$ and bw. All the relevant data were collected from published references. Dimensional analysis suggested VO_2max_ be proportional to $bw^{\frac{2}{3}}$. Statistics analysis displayed that four mathematical expressions were VO_2max_ = 0.047bw (p<0.01, R^2^ = 0.68), VO_2max_ = 0.036bw+0.71 (p<0.01, R^2^ = 0.76), VO_2max_ = 0.10bw^0.82^ (p<0.01, R^2^ = 0.93) and VO_2max_ = 0.23bw^0.66^–0.48 (p<0.01, R^2^ = 0.81) respectively. Pearson correlation coefficient showed a significant moderately negative relation between $\frac{VO_{2max}}{bw}$ and bw (r = -0.42, p<0.01), while there was no correlation between $\frac{VO_{2max}}{bw^{0.82}}$ and bw (r = 0.066, p = 0.41). Although statistics analysis did not fully verify the theoretical result, both dimensional and statistics analysis suggested ratio scaling distort the relationship and power function be more appropriate to describe the relationship. Additionally, we hypothesized that lean mass, rather than body weight, plays a more essential role in eliminating the gap between theoretical and experimental b values, and is more appropriate to standardize VO_2max_, future studies can focus more on it.

## Introduction

Proposed by Hill and Lupton in 1923, maximum oxygen uptake (VO_2max_) is a “gold standard” to evaluate cardiopulmonary function, widely used in evaluating individuals’ fitness state, making training plans, estimating cardiovascular risks etc. [1–5]. At present, ratio scaling ($\frac{VO_{2max}}{bw}$), a commonly used approach reflecting relative VO_2max_, is not only widely used in human but also in animal trials when comparing distinctions of aerobic capacity between samples. However, this method might be inappropriate to describe the quantitative relationship between VO_2max_ and body weight due to some key defects of the method in theory.

Robinson initially related VO_2max_ and body weight by using ratio scaling to analyze the distinctions of oxygen uptake capacity among boys and proposed an approach of “controlling” for growth [1]. However, this method may mislead pediatric exercise physiology for nearly a century. Many scholars including Tanner unequivocally established that expressing VO_2max_ in ratio with body weight was fallacious since they found that the defects of ratio scaling (e.g., the assumption of zero Y-intercept is untenable) can distort the actual relationship between VO_2max_ and body weight and mislead the practical application in sports [1, 5–7]. Instead, many scholars proposed that simple allometric model [6, 8, 9] (namely, power function relationship, y = ax^b^) rather than ratio scaling is better to describe the relationship between VO_2max_ and body weight. Regarding to exponent b, Kleiber’s original analysis [9] revealed that the best-fit b value for mammals, representing the sum of the influence of multiple contributors to metabolism and control, should be $\frac{3}{4}$ rather than 1. Likewise, Sarrus and Lambert developed from “surface law” and “kinematic or biological similarity” respectively and found that b should be $\frac{2}{3}$ instead of 1 [3, 10]. However, there is yet no consensus on theoretical b value since both $\frac{2}{3}$ and $\frac{3}{4}$ laws are supported by many experimental studies [2, 8, 11–18], for example, Taylor et al. [12] conducted a research on wild and domestic mammals and found that the b values should be 0.79 (95%CI: 0.75–0.83) and 0.76 (95%CI: 0.68–0.85) respectively; Werneck et al. [13] recruited school-aged pubertal girls and found that b value should be 0.52 (95%CI: 0.37–0.67). Given that, scholars including Feldman mentioned that two theoretical b laws were reasonable but should be utilized in different scopes (e.g., intraspecific versus interspecific, homogeneous samples versus heterogeneous samples) [5, 6, 19].

Therefore, the main purpose of this study was to preliminarily explore the general rule of VO_2max_ and body weight from theoretical (dimensional analysis) and experimental perspectives (statistics analysis). The secondary purpose was to provide a reference or information of a more appropriate and correct approach to express relative VO_2max_ before applying into practice to compare the distinctions of aerobic capacity.

## Methods

### Literature search

Electronic searching was performed in PubMed, Elsevier, Springer database etc. to collect experimental data relating to VO_2max_ and bw in order to reprocess and reanalyze. Key words included “maximal/maximum oxygen uptake” OR “aerobic capacity” OR “cardiorespiratory fitness” OR “athletes” AND “maximal/maximum oxygen uptake”.

#### Inclusion criteria

Inclusion criteria comprised: 1. Samples were mentally and physically healthy without any diseases/physical defects; 2. The age of human subjects should not exceed 40 years old; 3. Both males and females were included; 4. VO_2max_ values were attained from cardiopulmonary exercise testing (continuous incremental test on motorized treadmills); 5. Original data for VO_2max_ and body weight were shown in literatures.

#### Exclusion criteria

Exclusion criteria contained: 1. Samples had diseases/physical defects such as obesity (BMI≥28) and disability etc.; 2. The age of human samples exceeded 40 years old); 3. Literatures without original data of VO_2max_ and body weight should be excluded. Table 1 presents eleven literatures included in this study with data shown in two significant digits and means±SD.

**Table 1:** Data on VO_2max_ and body weight.

Study(reference)	VO_2max_(L/min)	Body weight(kg)	N
1 [20]	4.23±0.87	81±11	14
2 [12]	2.4±2.8	67±87	27
3 [21]	3.70±0.70	65±15	2
4 [22]	2.45±0.28	51.7±1.4	8
5 [23]	4.45±0.32	71.3±4.8	8
6 [24]	3.88±0.56	72.2±6.3	26
7 [25]	3.8	80	1
8 [26]	3.591±0.090	52.7±1.0	5
9 [27]	2.4±1.0	55±17	60
10 [28]	2.68±0.59	67.0±5.5	8
	3.0±1.6	64±39	159

The demographic characters in this study were displayed in Table 2.

**Table 2:** Demographic characters.

Age (yrs)	Height (m)	BMI
23.6±9.3	1.66±0.15	21.2±3.3

### Dimensional analysis

Dimensional analysis is an essential research method in natural science, reflecting the general law of quantitative relationship between variables based on the form that all quantities must have. Namely, the results of dimensional analysis are universal and could be used without concerning different situations. Since all the quantitative questions cannot escape from three basic physical variables (mass, length and time), these three variables are chosen to be a basic dimension system named three-dimensional MTL system, where M is mass, L is length and T is time in physics.

In MTL system, due to the fact that these basic variables in essence, are the products of one dimensional variable L in different dimensions (time is one-dimensional variable, while mass is three-dimensional) [10, 13], then let L = λ, all the variables in MTL system can be transformed into an n-th power relation with λ:

$$M=L^{3}=T^{3}=\lambda^{3}$$ (1)

To remove dimensions:

$$\left[M^{\alpha_{1}}\cdot L^{\beta_{1}}\cdot T^{\gamma_{1}}\right]\propto {\left[M^{\alpha_{2}}\cdot L^{\beta_{2}}\cdot T^{\gamma_{2}}\right]}^{b}$$ (2)

Hence, b can be calculated based on expressions above:

$$b=\frac{3\alpha_{1}+\beta_{1}+\gamma_{1}}{3\alpha_{2}+\beta_{2}+\gamma_{3}}$$ (3)

Therefore, the theoretical analysis of the nature relationship between VO_2max_ and m was calculated according to expression (3).

### Statistic analysis

Ratio scaling is a traditional way to distinguish individuals’ aerobic capacity, it means that when VO_2max_ and bw satisfy ratio relationship, we have bw = x, VO_2max_ = y = kx according to expression (4). Then, due to the fact that $\bar{\left(\frac{y}{x}\right)}=\frac{\sum_{i=1}^{n}\frac{kx_{i}}{x_{i}}}{n}=k=\frac{\left(\frac{\sum_{i=1}^{n}kx_{i}}{n}\right)}{\left(\frac{\sum_{i=1}^{n}x_{i}}{n}\right)}=\frac{\bar{y}}{\bar{\bar{x}}}$ and based on the knowledge of Least Square Estimate (estimated line passes mean values), k value in this study was dependent on mean values of VO_2max_ and bw.


y=kx (4)

Besides, to explore the relationship between VO_2max_ and bw, and to verify the dimensional analysis and the reliability of ratio scaling, we set up another 3 different mathematic models to reanalyze data using linear or nonlinear regression (independent variable was bw while dependent variable was VO_2max_). The models were linear function, simple allometric model and full allometric model (expression (5), (6) and (7) respectively).


y=k’x+d (5)

$$y=ax^{b}$$ (6)

$$y=a’x^{b’}+c$$ (7)

As for expression (6), it was translated into linear relation (expression (8)) firstly by using natural logarithm before regression. Apart from expression (7) that was analyzed using nonlinear regression, expression (5) and (8) were analyzed using linear regression.


lny=lna+blnx (8)

Based on the results of regression analysis, Pearson Correlation Coefficient was used to analyze the relationship between variable $\frac{VO_{2max}}{bw^{b}}$ (when b took different values) and bw in order to explore whether ratio scaling ($\frac{VO_{2max}}{bw}$)could exclude the influence of body weight effectively. Magnitude-based inferences suggested by Hopkins [29] was used to analyze the differences of aerobic capacity between light and heavy weight sample groups when the relative VO_2max_ was expressed by ratio scaling or allometric model (light or heavy samples were defined based on the statistics analysis results of expressions (4) and (6)): Cohen’s D effect sizes (ES) were calculated to reflect the extent of difference and the inferences associated with the effects defined as trivial (<0.20), small (0.20–0.59), moderate (0.60–1.19), large (1.2–1.9), very large (2.0–3.9) and extremely large (≥4.0). Furthermore, such magnitude-based inferences about effects can be made more accurate and informative by qualifying them with probabilities so that researchers can more clearly informed how much probability of this difference effect exists: most unlikely<0.5%<very unlikely<5%<unlikely<25%<possibly<75%<likely<95%<very likely<99.5%<most likely (e.g., if a moderate difference between A and B occurs, Hopkins’ Magnitude-based inferences can further provide the possibility of the occurrence of this moderate difference, for example, 25%-75% of possibility means that moderate difference occurs possibly). All the data were handled in SPSS20.0 and Excel. Significance level was set at 0.05 (p<0.05), and the results should be quoted in two significant digits.

## Results and discussion

### Theoretical b values

Given that the units of VO_2max_ and body weight are L/min and kg separately, so their dimensional expressions are $\left[D_{L\;VO_{2max}}\right]=\left[M^{0}\cdot L^{3}\cdot T^{-1}\right]$ and [*D*_*L bw*_] = [*M*^1^ ∙ *L*^0^ ∙ *T*^0^]. Therefore, based on Eq (3), $b=\frac{2}{3}$. The result suggests that VO_2max_ should be proportional to $bw^{\frac{2}{3}}$ (VO_2max_∝$bw^{\frac{2}{3}}$) instead of *bw*^1^, which is consistent with the results of Sarrus’s “surface law”, Lambert’s “kinematic or biological similarity” and West’s fractal geometry [3, 10, 30].

As mentioned above, dimensional analysis reflects the basic mathematical law between the variables. Therefore, from a strict dimensional analysis perspective, $\frac{2}{3}$ law should satisfy various experimental situations when expressing relative VO_2max_ to compare individuals’ aerobic capacity. However, not all the studies have supported $\frac{2}{3}$ law [2, 3, 6, 12]. Given that, some experts take mixed view on whether $\frac{2}{3}$ law could be a universal law and deduced b value from different theories, for instance, Kleiber [9] deemed that the optimal b value for mammals should be $\frac{3}{4}$ according to his original analysis. Likewise, McMahon considered that a muscle’s power output is only decided by its cross-sectional area A, while its shortening velocity ($\frac{\Delta l}{\Delta T}$) and tensile strength ($\sigma =\frac{F}{A}$) are constant [10]. Besides, based on the fact that area is a function of squared diameter, he deduced that maximal power output should be proportional to ${\left(m^{\frac{3}{8}}\right)}^{2}=m^{\frac{3}{4}}$. West et al. [30] considered that the energy and resources transport in the body should satisfy fractal geometry and got the same result by setting up a model based on animal cardiovascular system, respiratory system and plant vascular system. The reliability of $\frac{3}{4}$ law derived from these theories has been supported by many animal and human experiments [2, 12, 21]. Hence, in regard to $\frac{2}{3}$ and $\frac{3}{4}$ laws, Feldman and Heil et al. gave a different viewpoint and stated that both of them are rational theoretically but should be applied based on different conditions: $\frac{2}{3}$ law is more appropriate for within-species studies or homogeneous samples (age, height, background, etc.), whereas $\frac{3}{4}$ law should be applied to between-species studies or heterogeneous samples [5, 6, 19].

### Experimental b values

#### Ratio scaling and linear function

The estimated values for different models are given in Table 3. In ratio scaling the parameter k is 0.047 (95%CI:0.045–0.049), so Eq (4) now is VO_2max_ = 0.047bw (R^2^ = 0.68), whereas the parameters k’ and d in linear function are 0.036 (95%CI: 0.032–0.039) and 0.71 (95%CI: 0.47–0.95) respectively, so Eq (5) now is VO_2max_ = 0.036bw+0.71 (R^2^ = 0.76). If ratio scaling is reliable, this function should cross original point in coordinate system (namle, zero Y-intercept). However, given that the model R^2^ of linear function(R^2^ = 0.76) is better than that of ratio scaling (R^2^ = 0.68) and 95%CI of d value (95%CI: 0.47–0.95) does not include zero, the relationship between VO_2max_ and bw does not satisfy special linear function with d equal to zero. In other words, ratio scaling with zero Y-intercept assumption is not reliable, covering or misrepresenting the actual linear relationship between VO_2max_ and body weight.

**Table 3:** Estimated values for different models.

Models	Parameters		Standard error	p	95%CI		R^2^
					Lower limits	Upper limits	
y = kx	k	0.047	0.0010	<0.01	0.045	0.049	0.68
y = k’x+d	k’	0.036	0.0020	<0.01	0.032	0.039	0.76
	d	0.71	0.12	<0.01	0.47	0.95	
y = ax^b^	a	0.10	0.071	<0.01	0.087	0.11	0.93
	b	0.82	0.018	<0.01	0.78	0.85	
y = a’x^b^’+c	a’	0.23	0.065		0.11	0.36	0.81
	b’	0.66	0.050		0.56	0.76	
	c	-0.48	0.26		-0.99	0.026	

CI = confidence interval.

One of the potential reasons for the occurrence of zero Y-intercept assumption in ratio scaling, we assume, is over-prediction beyond the collected data range because most of the previous studies were human experiments in which subjects’ body weight mainly ranged from 50-100kg [31]. In other words, data of body weight outside that interval (especially less than 50kg) and its corresponding absolute VO_2max_ data were missing, causing the occurrence of over-prediction or zero Y-intercept in the relationship analysis between VO_2max_ and body weight when performing linear regression analysis based on the limited data range. In this study, we compensated for the defect by extending the data range (mainly ranging from 0-50kg), and the results support our viewpoint.

Furthermore, given that ratio scaling mirrors a new physical quantity defined by the ratio of two basic physical quantities, which is not affected by these two basic quantities, both sides of the Eq (5) are divided by x:

$$\frac{y}{x}=k'+\frac{d}{x}$$ (9)

It implies that if and only if d = 0, VO_2max_ and body weight satisfy ratio scaling ($\frac{VO_{2max}}{bw}$ is not restricted by bw), otherwise, $\frac{VO_{2max}}{bw}$ will decrease or increase based on the rise of independent variable bw and close to the line y = k when d>0 or d<0. Table 4 gives the results of Pearson Correlation Coefficient on the relationship between $\frac{VO_{2max}}{bw}$ and bw, showing that a significant moderately strong negative relationship exists between the variables (r = -0.42, p<0.01). It coincides with the results of Welsman and many other scholars [5, 19]. Therefore, relative VO_2max_ described by ratio scaling cannot escape from the influence of body weight.

**Table 4:** Pearson Correlation Coefficient of the relationship between $\frac{VO_{2max}}{bw}$ or $\frac{VO_{2max}}{bw^{0.82}}$ and body weight.

Variables`	Bw	
	r	P
$\frac{VO_{2max}}{bw}$	-0.42	<0.01
$\frac{VO_{2max}}{bw^{0.82}}$	0.066	0.41

There is a significant moderately strong negative relationship in $\frac{VO_{2max}}{bw}$ and bw (r = -0.42, p<0.01), while no correlation has shown in $\frac{VO_{2max}}{bw^{0.82}}$ and bw (r = -0.066, p = 0.41).

In summary, although both ratio scaling and linear function describe a linear relationship between the variables, they are quite different: based on the Least Square Estimate we know that the linear regression passes through the average point of data ($\bar{x},\bar{y}$) in coordinate system, which means that there is only one crossover point (the mean values) between ratio scaling and linear function. By comparison, linear function is better to describe the relationship between VO_2max_ and bw, whereas ratio scaling distorts the simple linear relationship between the variables because it ignores d≠0 which would cause large errors and mislead sports practice when evaluating the distinctions of aerobic capacity among individuals.

#### Ratio scaling and allometric models

Table 3 indicates that parameters a and b in simple allometric model are 0.10 (95%CI:0.087–0.11) and 0.82 (95%CI:0.76–0.91) respectively, so Eq (6) now is VO_2max_ = 0.10bw^0.82^ (R^2^ = 0.93). Meanwhile, parameters a’, b’ and c in full allometric model are 0.23 (95%CI:0.11–0.36), 0.66 (95%CI:0.56–0.76) and -0.48(95%CI:-0.99–0.026), so Eq (7) now is VO_2max_ = 0.23bw^0.66^–0.48 (R^2^ = 0.81). Therefore, two allometric models are better than the two linear models when describing the relationship between VO_2max_ and bw, and bw exponents in two allometric models are less than 1 with 95%CI excluding 1, which is consistent with the results of Werneck and many other scholars [6, 8, 12, 13, 21]. Therefore, the relationship between VO_2max_ and bw is not special linear but power function relationship. Besides, since the model R^2^ of simple allometric model is nearly perfect (R^2^ = 0.93) and Pearson Correlation Coefficient verifies that no correlation exists between $\frac{VO_{2max}}{bw^{0.82}}$ and bw (r = 0.066, p = 0.41), the best method to describe the relationship between VO_2max_ and body weight in this study is simple allometric model which is capable of eliminating the influence of body weight to VO_2max_.

**Fig 1:**
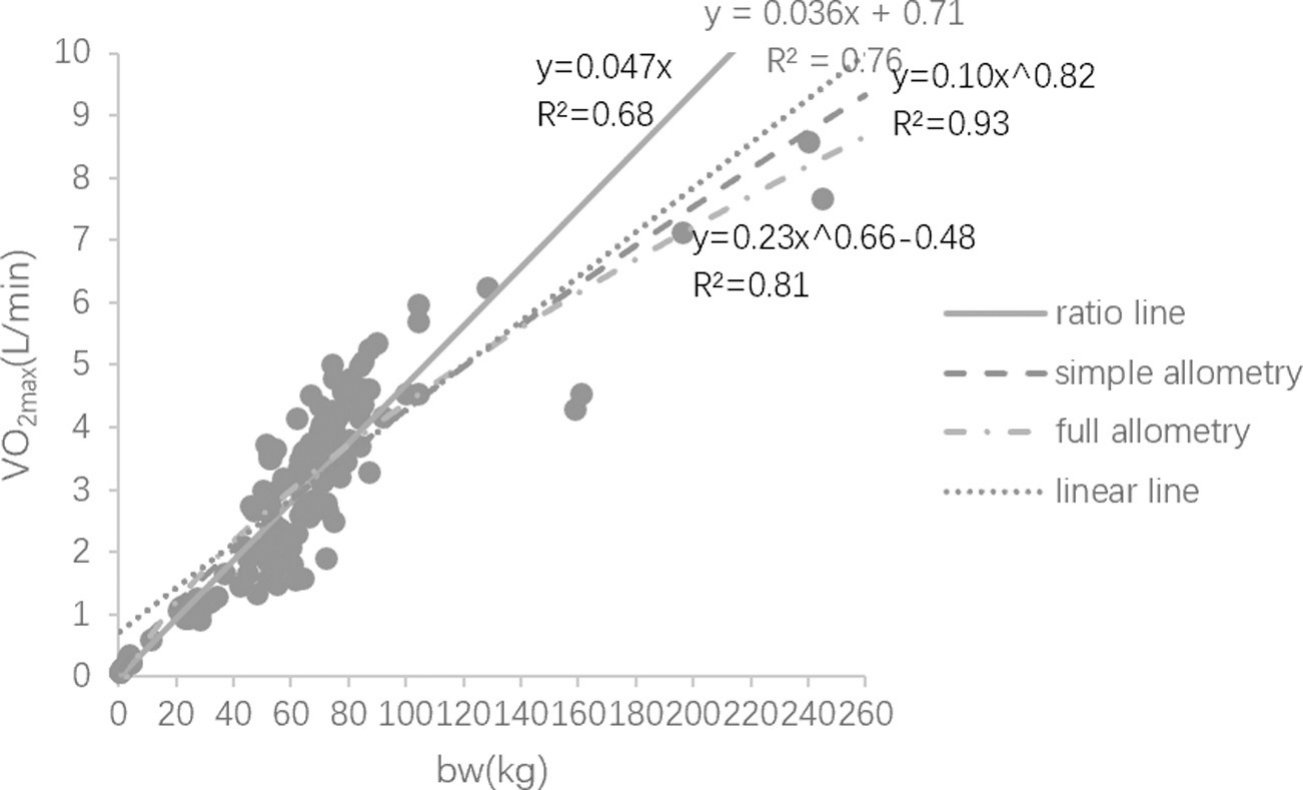
Data distribution and trends in different models.

**Fig 2:**
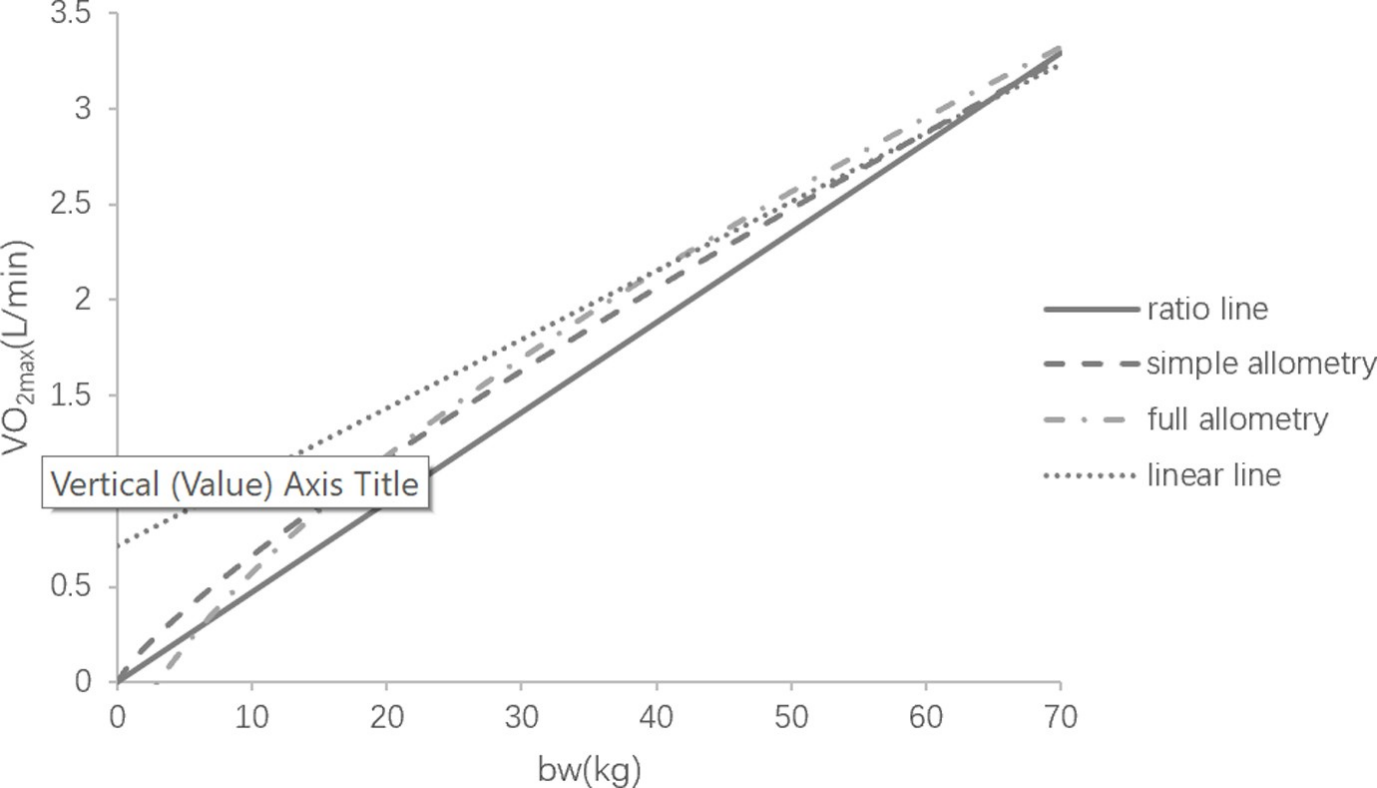
Trends of different models in the interval [0, 70].

Considerable evidence has shown that ratio scaling is not suitable to be used in expressing VO_2max_ and body weight’s relationship due to defects in theory, mathematics, etc., distorting the real relationship between the variables and misleading practical application in sports area. Fig 1 displays the distribution of the sample data in this study and change trends of the variables depicted by different models. Fig 2 displays the trends of four models more clearly in the interval [0, 70] by removing sample data. With respect of ratio scaling and simple allometric model, the two functions intersect only when x = 0 and x≈61 in the interval [0,+∞). Fig 1 (or Fig 2) illustrates that simple allometric model is above the ratio scaling in the interval (0,61), but it is completely opposite in the interval (61,260]. Given that the body weight of many athletes is 50-100kg, many errors may be caused if ratio scaling is applied to sports training, for instance, the aerobic capacity of the athletes with lighter body weight (e.g., young athletes) might be underestimated, whereas that of relatively heavy athletes might be overestimated. If this fake information is given to athletes and coaches, there is no doubt that it will affect the plans for the following training (e.g., aerobic capacity has been developed or it is no longer a main factor limiting the improvement). Apart from that, running capacity of the athletes with lighter body weight is likely underestimated to small extent when using ratio scaling [2], whereas it is most likely that those lighter samples are moderately superior in running capacity when evaluated by simple allometric model (according to Table 5). This finding is supported by Chamari [21] who conducted the research on the aerobic capacity of adult and young soccer players and gave the similar result, pointing out that the running capacity of young soccer player was worse than that of the adult players when using ratio scaling to evaluate, whereas that was not the case when using simple allometric model. As a result, coaches may mistakenly focus on refining running techniques at this stage if using ratio scaling.

**Table 5:** Relative VO_2max_ expressed by different models in subjects with light or heavy body weight.

	$\frac{VO_{2max}}{bw}$	$\frac{VO_{2max}}{bw^{0.82}}$
Light subjects (no more than 61kg, N = 71)	0.055±0.027	0.093±0.023
Heavy subjects (heavier than 61kg, N = 88)	0.049±0.0098	0.11±0.020
ES±95%CI	-0.41±0.46**	0.70±0.32****

In summary, ratio scaling distorts the relationship between VO_2max_ and bw due to its shortages in theory or mathematics, causing some problems including underestimating running capacity or overestimating aerobic capacity, and confusing the primary and the secondary problems when being applied to sports practice. In contrast, simple allometric model plays a very vital role in guiding sports training, evaluating sports skills or aerobic capacity correctly not only because it fits the theory, but also it seems to satisfy the reality better (e.g., elite marathon athletes always own relatively light body weight [32]) and reflects the real condition of athletes more correctly [11, 13].

Table 5 illustrates the mean relative VO_2max_ expressed by ratio scaling and simple allometric model in light or heavy subjects in this study. It is likely that lighter subjects show a greater value to small extent when using ratio scaling, whereas it is most likely that lighter subjects are moderately inferior in relative VO_2max_ when using simple allometric model. * means possibly (25–75%), ** means likely (75–95%), *** means very likely (95–99.5%) and **** means most likely (>99.5%). ES: trivial (<0.20), small (0.20–0.59), moderate (0.60–1.19), large (1.2–1.9), very large (2.0–3.9) and extremely large (≥4.0). Relative VO_2max_ values are shown in means±SD.

### Simple allometric model

#### The theory and meaning of simple allometric model

In the early 1930s, Huxley [33] found that an allometric equation y = ax^b^ could be used to describe biological functions and body dimensions when he studied the relationship between fiddler crabs’ claw size and body size. In fact, many interpretations in exercise sciences are empirical rather than theoretical derivation. Therefore, it is valuable to look insight to the quantitative relationship between VO_2max_ and body weight from a mathematic perspective, which may provide theory supporting and “insight into the biological design of the class” [6].

In a system where elements interact, the “growth” of the system is directly proportional to the number of influence factors [10]:

$$\frac{dE}{dt}=a_{1}\cdot E$$ (10)

Whether the growth of the system is positive or negative depends on whether the parameter *a*_1_ is positive or negative, When the parameter *a*_1_ is positive, the system increases as a whole, whereas when *a*_1_ is negative, it decreases. To integrate Eq (10), we have:

$$E=E_{0}\cdot e^{a_{1}t}$$ (11)

where *E*_0_ = *lnE*_0_. Eq (11) is a law of power function, also well-known as the “law of natural growth”, widely used in many fields. Besides, it can also be used for the study between different system parts. Based on Eq (10), if VO_2max_ and body weight are two independent systems, we have:

$$\frac{dE_{1}}{dt}=a_{1}\cdot E_{1}$$ (12)

$$\frac{dE_{2}}{dt}=a_{2}\cdot E_{2}$$ (13)

To integrate them and eliminate variable t:

$$E_{1}=\left(\frac{E_{10}}{{E_{20}}^{\frac{a_{1}}{a_{2}}}}\right)\cdot E_{2}{}^{\frac{a_{1}}{a_{2}}}$$ (14)

We define $\left(\frac{E_{10}}{E_{20}{}^{\frac{a_{1}}{a_{2}}}}\right)=a,\frac{a_{1}}{a_{2}}=b$ to simplify Eq (14):

$$E_{1}=a\cdot E_{2}{}^{b}$$ (15)

Eq (15) is the theoretical supporting of simple allometric model used to explore the quantitative relationship between VO_2max_ and body weight. In simple allometric model, the “growth” of the system is dependent on *a* (positive or negative), while b implies the complexity of the growing procedure in the system which is of critical interest [34]. When b>1 or b<1, the change of y is disproportionally faster or slower than the change of x (heterogony). When b = 1, their change is in sync (isogony). Therefore, to some extent, the widely used ratio scaling to describe quantitative relationship between VO_2max_ and body weight is significant from theoretical point of view since it is a special case of simple allometric model. However, this special case seems to be untenable since the result of statistics analysis using simple allometric model in this study did not support b = 1.

#### Shortages of simple allometric model

Although there are loads of defects, ratio scaling is still the first choice to evaluate the relationship between VO_2max_ and body weight. One of the potential reasons is its convenience. Compared with ratio scaling, simple allometric model is much more complex in calculation. From Figs 1 and 2 we know that the weight of the majority of subjects ranges from 50 to 100kg, and both ratio scaling and simple allometric model are quite similar in shape and almost coincide with each other in this range. The alteration of curvature |Δ*K*| in simple allometric model supports our visual feeling (Eqs (16) and (17)), suggesting that the line depicted by simple allometric model in this range nearly can be seemed as a straight line coincident with that depicted by ratio scaling.

$$K=\frac{\left|ab\left(b-1\right)\cdot x^{b-2}\right|}{{\left(1+{\left(ab\cdot x^{b-1}\right)}^{2}\right)}^{\frac{3}{2}}}$$ (16)

$$\left|\Delta K\right|=\left|K_{100}-K_{50}\right|\approx 0.000081\approx 0$$ (17)

By further calculation we noticed that the maximum difference between ratio scaling and simple allometric model is only 8.7% (which in fact could induce large errors). Therefore, ratio scaling is better to be used when considering from the perspective of convenience.

Apart from that, the difference between theoretical b values and the difference between theoretical and experimental b values are other two more essential reasons. Although we deduced the same theoretical b value from a dimensional perspective as some scholars (b = $\frac{2}{3}$), others got a different result when deducing from different theories (b = $\frac{3}{4}$). Regarding to these two theoretical values, some scholars proposed that $\frac{3}{4}$ law would be more appropriate to be a universal law than $\frac{2}{3}$ law. In fact, this argument has confused researchers for a long time because both of the laws are supported by considerable experimental evidence [35]. According to regression results in this study, $\frac{3}{4}$ law seems to be more reasonable than $\frac{2}{3}$ law since the difference between $\frac{2}{3}$ law and the experimental value is obvious. In order to further explore the relatively obvious difference in this study, five potential reasons are summarized and discussed bellowed.

Firstly, theoretical b value deduced from a strict dimensional analysis in this study should be generally appliable irrespective of the influence of samples themselves (e.g., different species), but the regression results of simple allometric model suggest that it is not the case. The main reason might be attributed to dimensional analysis itself which is mainly limited by researchers’ understanding to the variables. Namely, researchers need to find out all the potential factors affecting those variables as much as possible before figuring out their regularity, and to further revise the model (e.g., VO_2max_ = bw^b^ (or lean mass^b^)·exp(a·body fat+c·age). Therefore, $\frac{2}{3}$ law deduced by dimensional analysis in this study might be only appliable to homogeneous samples since we did not take the potential factors into consideration. Due to the fact that samples in this study are heterogeneous, future study should further verify whether their differences [5, 6, 19] including body composition, gender or species etc. really can be affecting factors. Secondly, different criteria for VO_2max_ plateau might affect the reliability and precision of VO_2max_, and therefore affect the b value to some extent. The different criteria might be related to the low frequency of the plateau phenomenon [36, 37]. Thirdly, the data in this study are not evenly distributed enough. The data in most of the previous studies were concentrated in a certain range, which may cause over-prediction beyond the data range. Although we avoided this problem by extending the data range, the data are not evenly distributed enough, covering the mathematical law. Fourthly, body weight might not be appropriate to be an independent variable in simple allometric model. Lolli et al. [6, 19] suggested that fat-free mass might be more appropriate and b value might not satisfy $\frac{2}{3}$ or $\frac{3}{4}$ law. For lean mass, the power function was close to 1 in his study. Therefore, it is fair to hypothesize that dynamic mass exponent might exist due to varying proportions of fat mass. Finally, the choice of different allometric models might affect b values due to defects of models themselves. Batterham et al. [6] found that the experimental b value (b = 0.65,95%CI:0.59–0.71) was close to the theoretical one when using simple allometric model, however, a linear relationship was given by full allometric model (b = 1,95%CI:0.70–1.31, c = 1.13,95%CI:0.54–1.73), and full allometric model (R^2^ = 0.586) fitted the data better than simple allometric model (R^2^ = 0.583). Given that simple allometric model almost coincides with full allometric model except in the vicinity of origin, he believed that simple allometric model is untenable because it seems to be dragged to pass origin by an invisible force. However, we have to point out that the VO_2max_ data and the corresponding body weight data (<50kg) are missing in Batterham’s research, so the results should be skeptical. In fact, by compensating for the missing data, we found that simple allometric model actually is tenable because 95%CI of the c value in full allometric model in our study suggests that it is possible to pass through the origin (c = -0.48,95%CI: -0.99–0.026). However, different b values calculated by different allometric models really confuse us. Since body weight exponent b measures ‘the complex of physiological processes’, different b values calculated by different allometric models suggest different physiological complexity. Besides, the b value calculated by full allometric model should be similar to the one calculated by simple allometric model because the c value in full allometric model supports the zero Y-intercept assumption in simple allometric model.

In summary, reasons including convenience, differences between theoretical b values as well as the differences between theoretical and experimental b values are potential reasons affecting the application of simple allometric model. Among the reasons, different attitudes to b values are the key issues restricting the substitution for ratio scaling. Considering the analysis mentioned above and the work done by Economos [35] who pointed out that relationship between mammals’ weight and their size can differently affect animals with different body sizes and therefore may affect the b value, we hypothesized that the b value might not be a static value but dynamic ($\frac{2}{3}\leq \text{b}<1$), but deeper understanding and systematic studies of the relationship between VO_2max_ and body weight are necessary in the future studies (especially the information on lean mass is critical for assessment of the relationship between VO_2max_ and body weight).

## Conclusion

Defects of theories and mathematics in ratio scaling distort the real relationship between VO_2max_ and body weight. In fact, they should satisfy power function relationship instead of special linear relationship (ratio scaling), and simple allometric model should be used to evaluate their relationship though it is still not perfect enough due to some limitations (e.g., differences between theoretical b values, differences between theoretical and experimental b values). In the future studies, researchers should pay more attention to various reasons affecting b values, especially the contribution of fat to body weight which is critical to be discussed in order to set up the using scope and refine simple allometric model.
